# Retraction: Corrigendum: L-Arginine uptake by cationic amino acid transporter promotes intra-macrophage survival of *Leishmania donovani* by enhancing arginase-mediated polyamine synthesis

**DOI:** 10.3389/fimmu.2024.1474944

**Published:** 2024-08-14

**Authors:** Frontiers Editorial Office

Following publication, concerns were raised regarding the integrity of the images in the published figures.

The authors failed to provide a satisfactory explanation during the investigation, which was conducted in accordance with Frontiers’ policies. As a result, the data and conclusions of the article have been deemed unreliable and the article has been retracted. The authors do not agree to this retraction.

